# *NOD1* Is Associated With the Susceptibility of Pekin Duck Flock to Duck Hepatitis A Virus Genotype 3

**DOI:** 10.3389/fimmu.2021.766740

**Published:** 2021-10-20

**Authors:** Suyun Liang, Ming-Shan Wang, Bo Zhang, Yulong Feng, Jing Tang, Ming Xie, Wei Huang, Qi Zhang, Dabing Zhang, Shuisheng Hou

**Affiliations:** ^1^ Key Laboratory of Animal (Poultry) Genetics Breeding and Reproduction, Ministry of Agriculture and Rural Affairs, Institute of Animal Sciences, Chinese Academy of Agricultural Sciences, Beijing, China; ^2^ Key Laboratory of Animal Epidemiology of the Ministry of Agriculture, College of Veterinary Medicine, China Agricultural University, Beijing, China; ^3^ Howard Hughes Medical Institute, University of California Santa Cruz, Santa Cruz, CA, United States

**Keywords:** Pekin duck, DHAV-3, *NOD1*, genome-wide association studies, transcriptome

## Abstract

Duck viral hepatitis (DVH) is an acute, highly lethal infectious disease of ducklings that causes huge losses in the duck industry. Duck hepatitis A virus genotype 3 (DHAV-3) has been one of the most prevalent DVH pathogen in the Asian duck industry in recent years. Here, we investigated the genetic basis of the resistance and susceptibility of ducks to DVH by comparing the genomes and transcriptomes of a resistant Pekin duck flock (Z8) and a susceptible Pekin duck flock (SZ7). Our comparative genomic and transcriptomic analyses suggested that *NOD1* showed a strong signal of association with DVH susceptibility in ducks. Then, we found that *NOD1* showed a significant expression difference between the livers of susceptible and resistant individuals after infection with DHAV-3, with higher expression in the SZ7 flock. Furthermore, suppression and overexpression experiments showed that the number of DHAV-3 genomic copies in primary duck hepatocytes was influenced by the expression level of *NOD1*. In addition, *in situ* RNAscope analysis showed that the localization of *NOD1* and DHAV-3 in liver cells was consistent. Altogether, our data suggested that *NOD1* was likely associated with DHAV-3 susceptibility in ducks, which provides a target for future investigations of the pathogenesis of DVH.

## Introduction

Duck viral hepatitis (DVH) is one of the most serious infectious diseases in Pekin ducks, as it may cause up to 90% mortality of ducks if not controlled (1). DVH can be caused by five agents, including duck hepatitis A virus genotypes 1 (DHAV-1), 2 (DHAV-2), and 3 (DHAV-3), which are members of the *Avihepatovirus* A species of the genus *Avihepatovirus* in the family *Picornavirida*e (2), and duck hepatitis virus type 2 (DHV-2) and duck hepatitis virus type 3 (DHV-3), which are classified within the genus *Avastrovirus* of the family *Astroviridae* (3). DHAV-3 has been the most prevalent pathogen of DVH in the Asian duck industry in recent years (4–7). To prevent DVH in ducklings, researchers aimed to develop effective vaccines against DHAV-3 in China (8, 9). However, the vaccines for DHAV-3 prevention are limited to the lab setting, and there is currently no licensed vaccine for the mass market. The lack of effective vaccines and complex pathogens increases the difficulty of DVH prevention and control.

Progress in poultry disease control (e.g., Marek’s disease) has proven that resistance breeding is an effective way to control infectious diseases (10–13). For the purpose of controlling DVH, resistance breeding against DHAV-3 was conducted in our poultry facility at the Institute of Animal Sciences, Chinese Academy of Agricultural Sciences. A resistant flock (Z8) of Pekin duck that displays significantly stronger resistance than other Pekin duck flocks was identified. Research on a host of factors related to the resistance or susceptibility of poultry to viral infection has made remarkable progress. The eukaryotic translation initiation factor 2 (eIF2) gene family may contribute to differential resistance to Newcastle disease virus in inbred Fayoumi and Leghorn lines (14). The duck possesses a contracted immune gene repertoire, and defense mechanisms against influenza infection in the duck have been optimized through the diversification of its β-defensin and butyrophilin-like repertoires (15). However, the differential responses to DHAV-3 infection in Pekin ducks from different genetic backgrounds are not yet known.

The innate immune system can improve host defense against pathogens (16). Pattern recognition receptors (PRRs) include membrane-bound Toll-like receptors (TLRs), RIG-like receptors (RLRs) and NOD-like receptors (NLRs), which can detect viruses. Recent reports suggest that *NOD1*, a member of the NLR family, could participate in coordinating host defense against viruses, as it might also respond to viral infections (17, 18). The conventional downstream effector molecule of *NOD1* is *RIPK2*, which initiates downstream signaling toward a variety of pathways, leading to MAPK and NF-κB signaling pathway activation and type I interferon (IFN-I) production (19–21).

To understand the genetic mechanisms that determine the differences in resistance or susceptibility to DHAV-3, two Pekin duck flocks, a resistant flock with an extremely low mortality rate and a susceptible flock (SZ7) with high mortality, were used to perform an infection experiment. Using Z8 and SZ7 flocks as models, genome-wide association studies and liver transcriptome analysis were conducted to detect the candidate genes responsible for DHAV-3 resistance. Since *NOD1* was shown to be responsible for the differences between resistance and susceptibility, we assessed the association between the expression of *NOD1* and the DHAV-3 genome.

## Materials and Methods

### Ethics Statement

The protocols involving animals were approved by the Animal Welfare and Ethics Committee of the Institute of Animal Sciences (IAS), Chinese Academy of Agricultural Sciences (IAS20160401, CAAS, Beijing, China).

### Virus

The DHAV-3 112803 isolate was originally isolated from a 1-week-old Pekin duckling showing clinical signs and pathological changes typical of DVH in 2011 in China and was stored at -80°C. The stock viral titer was determined in embryonating Pekin duck eggs, and the titer was 10^5.8^ ELD_50_/0.2 mL.

### Animals

A line of Pekin ducks, designated Z8; a susceptible flock of Pekin ducks, designated SZ7; and a control group, designated CON, were used in the study. The ducks were kept on the Pekin duck breeding farm of the Institute of Animal Sciences, Chinese Academy of Agricultural Sciences, Beijing, China.

### Cells

Primary duck hepatocytes were prepared from 17-day-old Pekin duck embryos *via* a method described by Woolcock (22) and were then maintained in growth medium consisting of DMEM supplemented with 10% fetal calf serum (FCS) and maintenance medium consisting of DMEM supplemented with 2% FCS (Corning, NY, USA). In addition, 100 U/ml penicillin and 0.1 mg/ml streptomycin were added to the medium. The cells were incubated at 37°C in 5% CO_2_ until use.

### Animal Experiment Design

The experiment included two intramuscular inoculation groups, consisting of 925 ducks from the Z8 flock and 272 ducks from the SZ7 flock, and one uninfected control group, comprising 9 ducks from the CON flock. We note that the Z8 flock shows excellent resistance to DHAV-3 while the SZ7 flock is susceptible to DHAV-3 and shows high mortality rate. So in the animal infection experiment, we had a greater number of laying Z8 ducks than SZ7 ducks. As the result, we obtained more eggs from Z8, which were all used for hatching at the same time. Therefore, the number of Z8 used in animal infection experiment is larger than that of SZ7. The ducks were marked with wing-tag according to their pedigree information. When the ducks reached 6 days of age, blood samples were collected from each duck and kept in tubes containing anticoagulants. At 7 days of age, ducks in both the Z8 and SZ7 groups were inoculated intramuscularly with 0.5 ml (10^5.8^ ELD_50_/bird) of DHAV-3 112803, and those in CON were inoculated intramuscularly with 0.5 ml of PBS. The mortality is observed every 2 h during the 12-48 hours postinoculation (hpi), and every 6 h during the 48-60 hours postinoculation (hpi). The dead ducklings were immediately examined for lesions.

We collected more than 30 blood samples for each of Z8 and SZ7 flock at 0, 1, 6, 12 and 24 hpi, respectively. We randomly picked twenty blood samples from each of Z8 and SZ7 to detect the virus load and four blood samples from each of Z8 and SZ7 for plasma isolation. Nine liver samples from each of the SZ7, CON, Z8-R (resistant ducks from Z8) and Z8-S (susceptible ducks from Z8) groups were collected at 24 hpi, which were all used for qPCR, western blotting and RNA *in situ* hybridization, respectively.

When the surviving ducks in the experimental groups described above reached 42 days of age, 30 ducks (half male, half female) from each of the Z8 and SZ7 groups were selected for slaughter tests. The following performance indices were recorded: live weight, chest muscle weight, leg muscle weight, sebum weight, abdominal fat weight, carcass weight, chest width, and keel length. For the each of surviving birds, when egg production reached a peak at approximately 30 to 40 weeks of age, the egg-laying rate and hatching rate were recorded.

### Plasma Biochemistry Analysis

Plasma was harvested and tested for biochemical markers, including alanine aminotransferase (ALT), aspartate aminotransferase (AST) and alkaline phosphatase (ALP). Biochemistry analysis was conducted using a fully automatic biochemical analyzer (Hitachi 7080, Japan).

### Resequencing of the Duck Genome

A total of 110 blood samples were selected for DNA extraction using the phenol-chloroform protocol, including 50 Z8-R ducks, 10 Z8-S ducks, and 50 dead duck samples from SZ7 (SZ7-S). Two paired-end libraries with an insert size of 300 bp were constructed according to the Illumina manufacturer’s instructions and subjected to PE150 sequencing on the HiSeq 2500 sequencing platform (Illumina, San Diego, CA). Our methodology and the procedure for detecting mutations were described previously (23).

### Genome-Wide Association Studies

The population structure and cryptic relationships were considered to minimize false-positive results. The mixed linear model program EMMAX (24) was used for association analysis. with setting “emmax -v -d 10 -t test_sort.vcf -p test.trait.txt -k kinship -c pca.txt -o out.txt”. We defined the whole-genome significance cutoff as the Bonferroni test threshold, and we set the association threshold as 0.01/total SNPs (−log_10_ (P) = 9.20). Further, two other software TASSLE version 5.0 (25) and FaST-LMM version 0.2.33 (26) were used for verification. GLM and MLM models in the TASSEL were association analyses respectively. FaST-LMM was used for association analysis with setting “fastlmmc -tfile test_sort.vcf -pheno test.trait.txt -tfileSim test.impute -simOut test_sim -out T01HR1.pvalue -missingPhenotype NA”.

### Screening for Candidate Regions

To define candidate regions that have undergone directional selection during domestication, the population-differentiation statistic (*F_ST_*) and nucleotide diversity (π) were calculated using the program is Vcftools version 0.1.15 (27). These calculations were performed using 50 kb sliding windows with a 25 kb step and a minimum allele frequency (MAF) of 0.05, and unrelated individuals were selected for analysis (28). For calculating the *F_ST_*, we used the command “vcftools –vcf input_data.vcf –weir-fst-pop pop_1.txt –weir-fst-pop pop_2.txt –max-missing 0.9 –maf 0.05 –fst-window-size 50000 –fst-window-step 25000 –out pop1_vs_pop2”, and for calculating π, we used the command “vcftools –vcf pop1.recode.vcf –max-missing 0.9 –maf 0.05 –window-pi 10000 –window-pi-step 5000 –out pop1.recode.vcf.pi”. All genotyped SNPs were color coded according to their pairwise LD with the leader SNP obtained by GWAS.

### Transcriptome Analysis

For the susceptibility of Z8-S was more representative than other groups, then liver samples were collected from 9 Z8-R ducks and 9 Z8-S ducks for transcriptomic analysis. RNA was extracted from 200 µl of supernatant by using the TRIzol method. The methods of transcriptome sequencing and RNA-seq data analysis were the same as those described in our previous study (29). The initial RNA-seq sequences were processed by trimming of primers and filtration of low-quality reads using NGS QC Toolkit version 2.3.3 (30). The filtered sequences were mapped to the known genome sequence of Anas platyrhynchos (assembly IASCAAS_Pekin Duck_PBH1.5) using TOPHAT v2.0.13 (31) with setting “tophat2 -r 50 -p 8IASCAAS_Pekin Duck_PBH1.5 *_R1.fastq.gz *_R2.fastq.gz -o tophatotput”. The CUFFLINKS v2.2.1 software (32) was applied to assembly and calculate FPKM with setting “cufflinks -p 8-GIASCAAS_Pekin Duck_PBH1.5.gtf -o/geneexp -b IASCAAS_Pekin Duck_PBH1.5.fasta accepted_hits.bam” and to screen differentially expressed genes (DEGs) with parameters log_2_FC > 2 or <-2, P < 0.05, and FDR < 0.01.

### Western Blotting

The tissue samples were ground, and the cells were lysed in an ice bath in protein extract solution. The cells were then sonicated and centrifuged at 4°C at 12000 rpm for 15 minutes, and the supernatant was collected. Protein concentrations were measured by using a bicinchoninic acid (BCA) protein assay kit (Pierce, MA, USA). The protein samples were separated by SDS-PAGE, transferred to PVDF membranes, blocked with PBST (PBS with 0.05% Tween-20) containing 5% milk for 1.5 h, and then probed with a rabbit anti-duck polyclonal antibody (Biodragon, Beijing, China) as the primary antibody. The membranes were then washed with PBST and probed with HRP-conjugated goat anti-rabbit IgG as the secondary antibody.

### Dose-Dependent Analysis

Primary duck hepatocytes from SZ7 embryos were used to explore the dose-dependent effects of DHAV-3 on the expression of related genes. A total of 1.4×10^5^ cells were seeded in 24-well plates. The cells were infected with DHAV-3 at multiplicities of infection (MOIs) of 1, 0.1, and 0.01 copies/cell. The embryo hepatocytes from the CON group were inoculated with PBS, which was used as control. The expression levels of related genes, including *NOD1, MAPK1, IL8* and *IFN-β*, were detected at 24 hpi. Samples were collected from three wells of 24-well plates, with each well being analyzed three times.

### Suppression and Overexpression of *NOD1*

Three different *NOD1*-specific target sequences were used for the knockdown of *NOD1*, and the sequences of the small interfering RNAs (siRNAs) are listed in **Supplementary Table S15**. γ-D-Glu-mDAP (iE-DAP) (InvivoGen, CA, USA), the minimal motif recognized by the intracellular receptor *NOD1*, was used to overexpress the mRNA. A total of 4×10^5^ cells were seeded in 12-well plates and infected with DHAV-3 at an MOI of 1 copy/cell. siRNA transfection and the treatment of cells with iE-DAP were performed according to the manufacturer’s instructions. The embryo hepatocytes from the CON group were inoculated with PBS and used as control.

### Dynamic Expression of *NOD1* and DHAV-3

Primary duck hepatocytes from the Z8 and SZ7 groups were grown in 24-well plates (2×10^5^ cells/well), washed three times with PBS and inoculated with DHAV-3 at an MOI of 1. The embryo hepatocytes from the CON group were inoculated with PBS and used as control. After inoculation, the medium containing the cells were sampled at 0, 12, 24, 36 and 48 hpi. The DHAV-3 qPCR assay was performed as described previously (33).

### RNAscope *In Situ* Hybridization

To detect viral RNA and *NOD1* mRNA, *in situ* hybridization was performed by using the RNAscope 2.5 HD Reagent kit-red (ACD, Newark, CA; Cat No: #322350). Single-gene *in situ* RNAscope transcripts are shown in red. Two 20ZZ probes, V-DHAV-3 targeting 652-1588 of GU066821.1 and Ap-*NOD1* targeting 802-1711 of NM_001310381.1, were designed by the ACD probe design team. RNAscope analysis was performed on 4% paraformaldehyde-fixed liver tissue according to the manufacturer’s protocol using probes against *NOD1* and DHAV-3.

### Real-Time Quantification PCR Analysis

Sample processing and RNA extraction were conducted as described above, and qPCR assays were applied to detect the expression of related genes, including *NOD1, IL8, IFN-γ, IFN-β, IL1-β, MAPK1, RIPK2, IKBKB, MAPK8, TRAF3, TBK1, IL18, IL6, IL23A, TNFAIP1, TNFAIP3 CCL20, CCL26, CCL22, CCL19* and *CCR7*. RT-qPCR was performed as described previously (29). The liver tissues and embryo hepatocytes from the CON group were used as the mock-infected group, and its mRNA level was taken as the baseline. The primers are listed in **Supplementary Table S16**.

### Statistical Analysis

Data obtained in animal experiments and RT-qPCR assays were expressed as mean values ± standard Error of Mean (SEM). Differences between groups were compared using Student’s t-tests in the GraphPad Prism 6.0 program. A *P* < 0.05 value was considered statistically significant.

## Results

### Phenotypic Characteristics of Z8 and SZ7 Flocks Infected With DHAV-3

**Figure 1:**
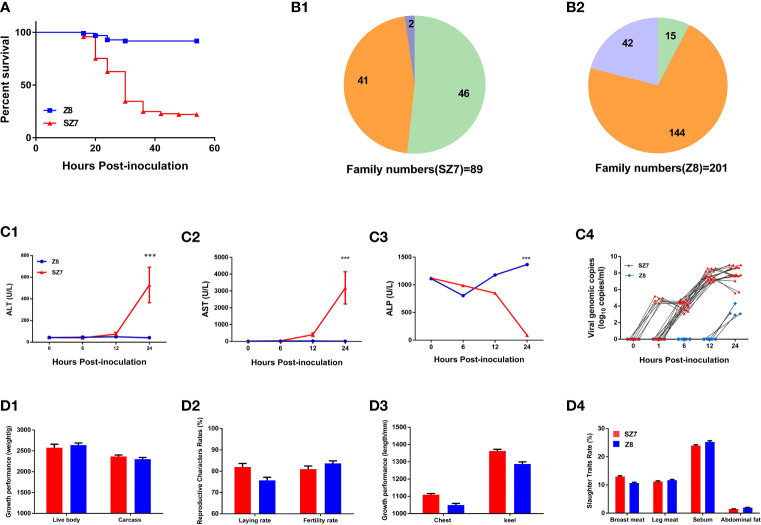
Phenotypic differences between the Z8 and SZ7 flocks infected with DHAV-3. **(A)** Survival curves of populations Z8 and SZ7 used in this experiment. The figure shows the death curve for the intramuscular route of infection. **(B1)** Numbers of SZ7 families showing 100% survival, 100% mortality, 33.3% mortality, and 66.6% mortality. **(B2)** Numbers of Z8 families showing 100% survival, 100% mortality, and 11.1%-88.9% mortality. Orange represents the proportion of families with a mortality rate of 0%, green represents the proportion of families with a mortality rate of 100%, and blue represents the proportion of families with a mortality rate of >0 - <100%. **(C)** Blood ALT **(C1)**, AST **(C2)**, ALP **(C3)** and viral load levels **(C4)** in the population Z8 and SZ7 ducklings used in this experiment. The serum samples at each time point include 4 individual data points. The viral load at each time point includes 20 individual data points. ***P < 0.001. **(D)** The growth and reproductive performance of Z8 (n = 30) and SZ7 (n = 30), including live weight and carcass weight **(D1)**, egg-laying rate and hatching rate **(D2)**, chest width and keel length **(D3)**, rate of breast meat, leg meat, sebum and abdominal fat **(D4)**.

We conducted experimental infections with DHAV-3, employing a susceptible flock of Pekin ducks as a control. After intramuscular inoculation, mortality occurred within 18-48 hpi, and the ducklings exhibited typical arch reflexes. The mortality rates of Z8 and SZ7 were 7.8% and 67.5%, respectively (**Figure 1A**). Among the 201 families in the Z8 group, deaths were observed in 57 families, with mortality rates of 100% in 15 families and 11.1%-88.9% in 42 families. Among the 89 families in the SZ7 group, mortality appeared in 48 families, with a death rate of 100% in 46 families and 33.3%-66.6% in 2 families. In the Z8 flock, 144/201 (71.6%) families showed strong resistance to DHAV-3, while in the SZ7 flock, 46/89 (51.7%) families exhibited high susceptibility (**Figure 1B**).

Plasma biochemical markers were detected to evaluate the degree of hepatic injury in the Z8 and SZ7 groups (**Supplementary Table S1**). The levels of ALT and AST in the plasma of flock SZ7 increased up to 12 hpi (**Figure 1C**) and were significantly different from the levels measured in flock Z8 (*P* < 0.001, the Student’s t-tests). The levels of ALP in the plasma of flock SZ7 were significantly different from those in Z8 at 24 hpi (*P* < 0.001, the Student’s t-tests). As a marker of DHAV-3 replication in the early host infection stages, we compared the difference in viremia between Z8 and SZ7. In flock SZ7, DHAV-3 was detected at 1, 6, 12, and 24 hpi. In contrast, DHAV-3 was not detected until 24 hpi in flock Z8 (**Figure 1C**). The viral loads in the blood samples of the two groups were significantly different at each sampling time (*P* < 0.001; the Student’s t-tests; **Supplementary Table S2**).

To investigate whether the growth and reproductive performance of the ducks were affected by resistance breeding, we measured a series of indices, including meat performance, body size, and reproductive performance. There were no significant differences in these measured indices between the Z8 and SZ7 flocks (*P* > 0.05, the Student’s t-tests; **Figure 1D**).

### Identification of Candidate Regions by Genome-Wide Analysis

**Figure 2:**
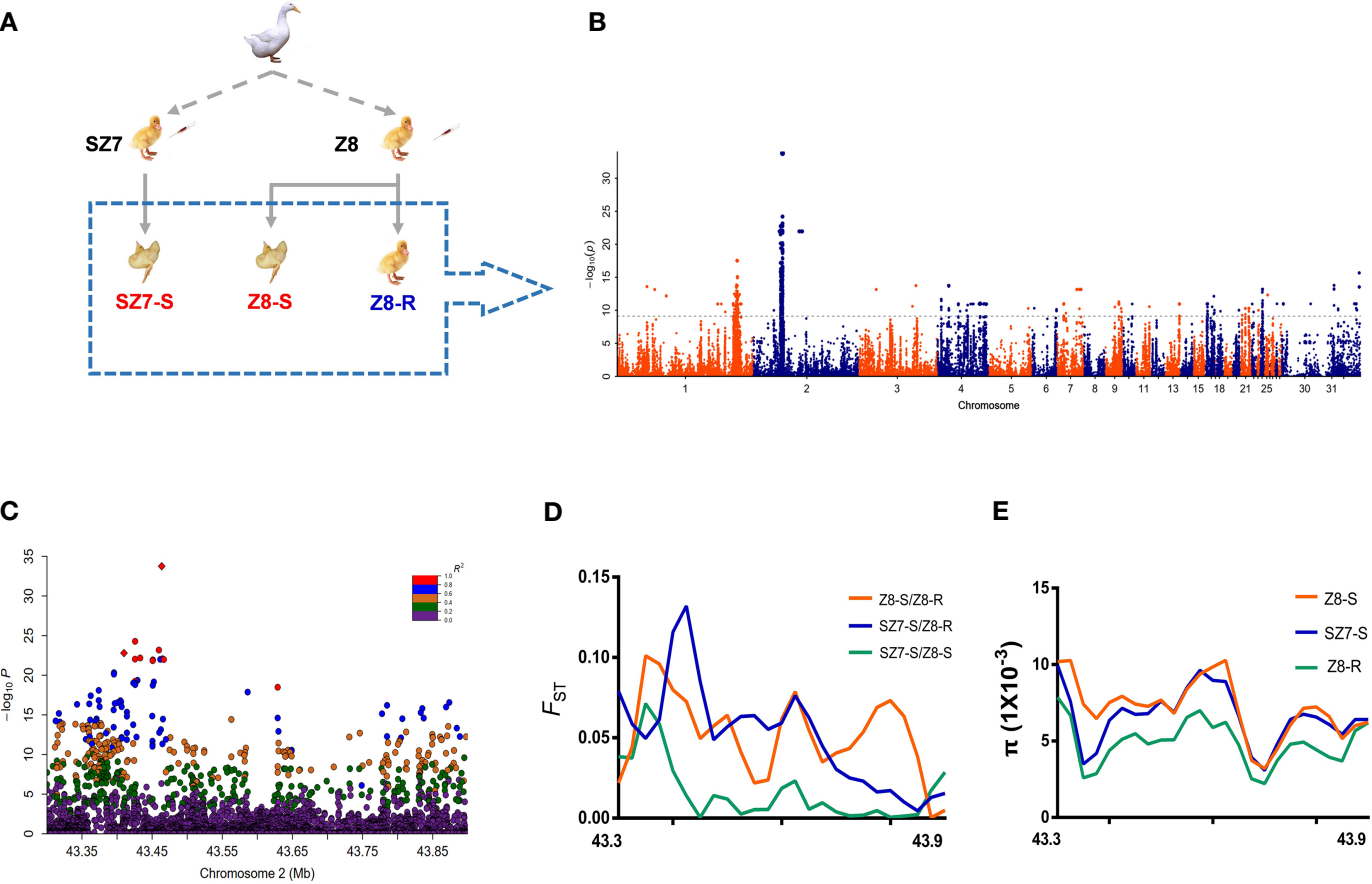
Analysis of genetic variation related to the susceptibility trait. **(A)** Images are from individuals of flock Z8 and flock SZ7. The dashed box shows the selection of animal materials used in this study for resequencing. **(B)** Manhattan plot showing the genetic effects on the susceptibility trait (observation color grade value) determined by GWAS in 110 Pekin ducks. The gray line represents the Bonferroni-corrected significance threshold (-log_10_ P = 9.20). **(C)** Regional plots of the loci ranging from 43.30 to 43.90 Mb along chromosome 2 associated with the susceptibility trait. All genotyped SNPs are color coded according to their pairwise linkage disequilibrium (LD) with the leader SNP (Chr2:43,445,923) calculated in the Pekin ducks. **(D)** Selective sweeps among three duck populations within the candidate region (Chr2:43.30-43.90 Mb). **(E)** The nucleotide diversity (π) of SZ7-S (blue line), Z8-R (green line) and Z8-S (red line) from 43.30 to 43.90 Mb on chromosome 2.

Based on the animal experiments described above, a total of 110 samples, including 50 live duck samples from the Z8 flock (Z8-R), 10 dead duck samples from Z8 (Z8-S), and 50 dead duck samples from SZ7 (SZ7-S) samples, were selected to perform whole-genome resequencing (**Figure 2A** and **Supplementary Table S3**). A total of 1.07 T of genomic data was generated (**Supplementary Table S4** and **Supplementary Figures S1, S2**) and the genomic sequencing depth for each sample was ~10-fold.

To identify candidate genes responsible for resistance or susceptibility, we conducted efficient mixed-model association, expedited for genome-wide association studies (**Supplementary Figures S3–S6** and **Supplementary Tables S5, S6**). A total of 22 regions were statistically significant, with the chr2:43.3-43.9 Mb region showing the strongest signal, followed by the chr1: 169.6-181.4 Mb region (**Figure 2B** and **Supplementary Table S7**). Similar patterns were observed in the other analyses (**Supplementary Figures S7–S9**).

To define candidate regions that have undergone selection during breeding, the population-differentiation statistic (*F_ST_*) and nucleotide diversity (π) were calculated. As expected, we observed that the chr2:43.30-43.90 Mb region showed extremely low levels of π and a higher *F_ST_* value in Z8-R than in the susceptible population (**Figures 2D, E** and **Supplementary Tables S9, S10**). In addition, we found that 195 SNPs spanning this region were highly correlated (pairwise r^2^ > 0.6, **Figure 2C** and **Supplementary Table S8**). Overall, the above analyses suggested that genes in the chr2:43.30-43.90 Mb region were likely to play a central role in Z8-R; thus, we focused on genes in this region in subsequent analyses.

### RNA-Seq Analysis of Candidate Genes

**Figure 3:**
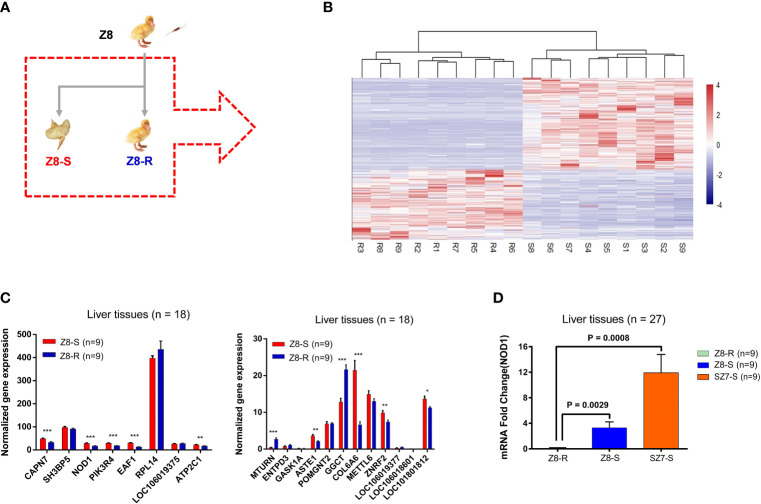
*NOD1* is a candidate gene for the susceptibility trait. **(A)** Images are from individuals of flock Z8. The images in the dashed box show the selection of animal materials used in this study for transcriptome sequencing. **(B)** Heat map analysis classifying the gene expression patterns of DEGs. **(C)** Gene expression levels in candidate intervals determined by the transcriptome analysis of liver samples. Statistical analysis was performed by using Student’s t-test, and error bars indicate the standard error of the mean (SEM). The results are presented as the means ± SEM of at least three independent experiments. *P < 0.05; **P < 0.01; ***P < 0.001. **(D)** qPCR was used to determine the expression of *NOD1* in liver tissues from Z8-R (n = 9), SZ7-S (n = 9) and Z8-S (n = 9) ducks.

Annotating the SNPs within chr2:43.30-43.90Mb did not reveal any mutation that changed the protein amino-acid sequence, suggesting that causative mutations are likely located in regulatory regions that affect gene expression levels (**Supplementary Tables S11, 12**). Therefore, we performed the mRNA-seq of liver tissues from both Z8-S (n=9) and Z8-R (n=9) (**Figure 3A**) to measure gene expression levels. A total of 2,841 genes showed a significant difference in their expression between the two groups (*P* < 0.01, corrected by FDR < 0.01, **Figure 3B** and **Supplementary Table S13**).

**Figure 4:**
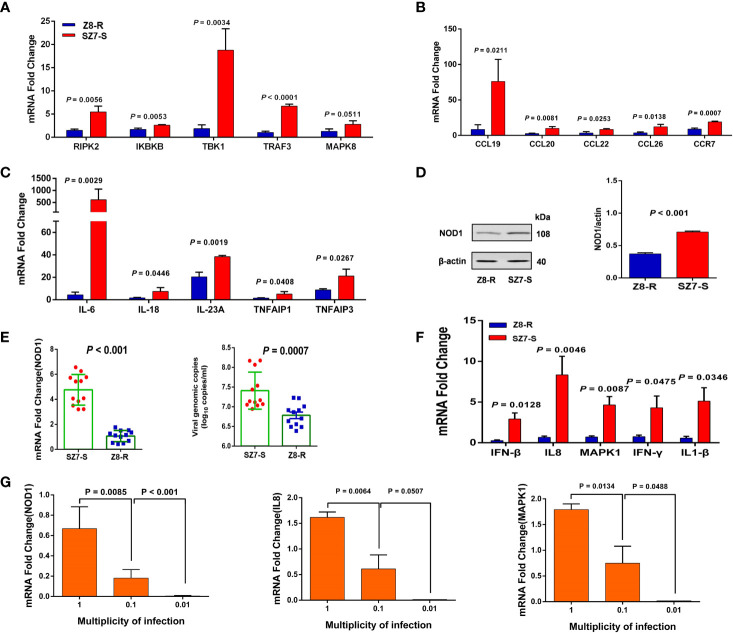
The expression of *NOD1* and downstream genes is activated by DHAV-3 infection. **(A)** RT**-**qPCR was used to quantify the expression of genes involved in *NOD1* signaling pathways. **(B)** RT**-**qPCR was used to determine the expression of chemokines. **(C)** RT**-**qPCR quantification of the expression of inflammatory factors. **(D)** The protein expression levels of *NOD1* in the livers of Z8-R and SZ7-S. Representative western blot (left panel) and quantification results from three independent experiments (right panels). **(E)** RT-qPCR quantification of the expression of *NOD1* and the genomic copy numbers of DHAV-3 in primary liver cells from Z8-R (n = 9) and SZ7-S (n = 9). **(F)** RT**-**qPCR analysis of *IL8, IFN-β, MAPK1, IFN-γ*, and *IL1-β* mRNAs in the liver cells of Z8-R and SZ7-S ducks. **(G)** The expression levels of *NOD1, IL8* and *MAPK1* at different multiplicities of infection. Primary liver cells of SZ7-S ducks were used in this experiment.

Among the 20 genes in the chr2:43.30-43.90 Mb region, 11 genes were expressed at significant levels. Interestingly, the expression of *NOD1*, a crucial gene in the NOD-like signaling pathway, was significantly downregulated in Z8-R relative to Z8-S (**Figure 3C**). Then, we performed qPCR to further evaluate the expression of *NOD1* in the liver samples of Z8-R, Z8-S and SZ7-S flocks after infection with DHAV-3. Consistent with the RNA-seq results, we found that the expression level of *NOD1* was significantly lower in Z8-R than in Z8-S and SZ7-S (*P* < 0.01, **Figure 3D**). Moreover, western blot analysis showed that the protein expression level of *NOD1* in SZ7-S was higher than that in Z8-R (**Figure 4D**). In addition, we measured the expression levels of downstream genes potentially regulated by *NOD1* (including genes involved in *NOD1* signaling pathways (*RIPK2, IKBKB, MAPK8, TRAF3* and *TBK1*), inflammatory factors (*IL18, IL6, IL23A, TNFAIP1* and *TNFAIP3*) and chemokines (*CCL20, CCL26, CCL22, CCL19* and *CCR7*)) and found that the expression levels of these downstream genes were significantly different between the Z8-R and SZ7-S groups (**Figures 4A–C** and **Supplementary Figure S10**, **Supplementary Table S14**).

### Analysis of the Expression of *NOD1* and Related Genes

To examine the specific role of *NOD1* in the response to DHAV-3 infection, primary duck hepatocytes from SZ7-S and Z8-R were cultured and infected with DHAV-3. The expression levels of *NOD1* and related genes (*IL8, IFN-β, MAPK1, IFN-γ*, and *IL1-β*) were examined. Consistent with the above analysis, the expression of *NOD1* was found to be significantly downregulated in Z8-R, and the viral load in the Z8-R flock was significantly lower than that in the SZ7-S flock at 24 hpi (**Figure 4E**). Interestingly, the expression levels of *IL8, IFN-β, MAPK1, IFN-γ* and *IL1-β* were also significantly downregulated in the livers of Z8-R ducks (**Figure 4F** and **Supplementary Figure S11**).

Next, we assessed the relevance of the host pathway to DHAV-3 infection using liver primary cells of SZ7-S ducks and examined the expression levels of *NOD1* after their inoculation at MOIs of 1, 0.1, and 0.01. DHAV-3 induced the expression of *NOD1* in a dose-dependent manner (**Figure 4G**). As expected, the expression levels of the related genes appeared to be consistent with that of *NOD1* (**Supplementary Figure S12**).

### Correlation Between DHAV-3 Replication and *NOD1* mRNA Expression

**Figure 5:**
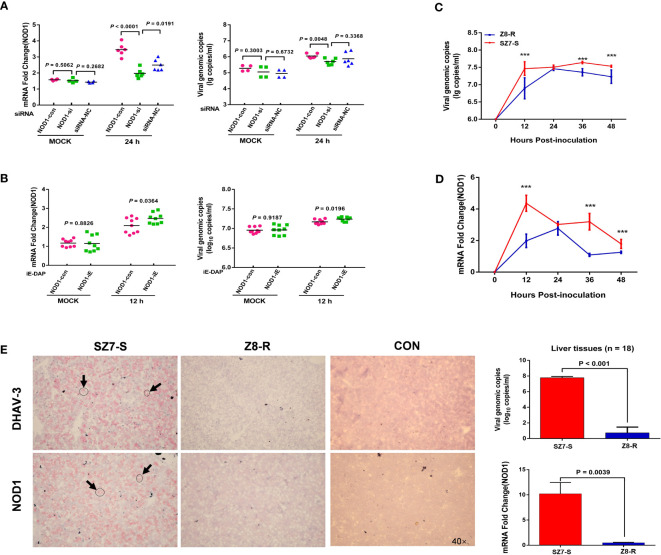
*NOD1* is correlated with DHAV-3 infection. **(A)** RT-qPCR analysis of *NOD1* and DHAV-3 mRNA expression in primary liver cells. Cells of the MOCK group were sampled at 12 hpi. **(B)** RT-qPCR analysis of *NOD1* and DHAV-3 mRNA expression in primary liver cells. Cells were treated with iE-DAP (50 μg/ml) for the indicated times. Cells of the MOCK group were sampled at 12 hpi. **(C, D)** The kinetics of DHAV-3 and *NOD1* expression in primary liver cells. The results are presented as the means ± SD of at least three independent experiments. ***P < 0.001. **(E)** DHAV-3 and *NOD1* were detected by the RNAscope *in situ* hybridization method, and RT-qPCR was used to determine the expression of *NOD1* and the genomic copy numbers of DHAV-3. Statistical analysis was performed by Student’s t-test, and error bars indicate the standard error of the mean (SEM). Images were acquired with an OLYMPUS microscope. Oil objective: 40×; zoom in 1×.

To investigate the effect of *NOD1* expression on DHAV-3 replication, a suppression experiment was conducted. At multiple time points, we monitored DHAV-3 genomic copy numbers and *NOD1* expression levels in two cell suspensions infected by the same amount of virus. At 12 hpi, there was no difference in the viral load or *NOD1* expression level between the two groups; after 24 hours of suppression, *NOD1* expression decreased significantly (*P* < 0.01), and the DHAV-3 genomic copy number also decreased significantly (*P* < 0.01, **Figure 5A**). The expression levels of downstream genes were also greatly altered after 24 hours (*P* < 0.05, **Supplementary Figure S13**). Furthermore, we performed a reverse experiment using iE-DAP to overexpress *NOD1* mRNA, the results were consistent with the results of the interference test, showing a significant increase in the DHAV-3 genomic copy number (*P* < 0.05, **Figure 5B**).

To investigate the role of *NOD1* in DHAV-3 infection in ducks, we determined the expression patterns of DHAV-3 and *NOD1* in cells. Our results showed that there was a rapid rise in the virus load in the SZ7-S group, which differed significantly from the load in the Z8-R group (*P* < 0.001, **Figure 5C**). In addition, there was a significant increase in the expression of *NOD1* in the SZ7-S group at 12 hpi, which was also notably different from the expression level of the Z8-R group (**Figure 5D** and **Supplementary Figure S14**).

Last, light microscopy showed that both *NOD1* and viral RNA (detected by RNAscope *in situ* hybridization) were widely expressed in the cytoplasm (**Figure 5E** and **Supplementary Figure S15**). Together, the above results suggest that *NOD1* likely plays a key role in the susceptibility trait correlated with viral RNA.

## Discussion

In the current research, we conducted comparative studies on the difference between Z8 and SZ7. The Z8 flock presented strong resistance against DHAV-3 infection following four generations of selection. The degree of liver damage (e.g., AST and ALT levels), apoptosis and virus replication efficiency (e.g., viremia) were also significantly different between the two flocks. However, we found that the Z8 flock exhibited similar productive and growth performance to SZ7, indicating that resistance breeding would only lead to changes in the Z8 resistance phenotype. The successful breeding of Z8 provided a good model and opportunity for genomic studies of the mechanisms of duck hepatitis virus infection.

In infection experiments, all ducks in 144 families of the Z8 flock survived virus infection, whereas 100% mortality appeared in 46 families of the SZ7 flock. This suggesting that the resistance of the Z8 and SZ7 Pekin ducks is strongly family correlated. The present observations support the view that it is feasible to construct a resistant line of Pekin ducks by using the strategy of family selection together with infection experiments (33). Furthermore, genetic factors have been shown to be closely related to viral hepatitis infection, and families will be an essential consideration in subsequent studies.

*NOD1*, an innate immune response-related gene, is a pattern recognition receptor regulating the expression of proinflammatory factors and interferons (34, 35). Our comparative genomic analysis suggests a strong association between the *NOD1* and resistance or susceptibility of DHAV-3 in ducks. Especially, the expression level of *NOD1* was significantly higher in the SZ7 flock than in the Z8 flock at 12 hpi. Our study showed that there was a correlation between *NOD1* and DHAV-3 and that the expression of *NOD1* was upregulated in susceptible ducks, which was consistent with the study by Vegna 2016. Previous studies also showed that a large number of cytokines and type I interferons are expressed during DHAV-3 infection in ducks (33, 36, 37). These important clues suggest that innate immunity is likely activated during infection with DHAV-3. We hypothesize that DHAV-3 infection likely trigger the *NOD1* signaling pathway, causing the type I interferon gene and cytokine expression levels to be significantly upregulated, which resulted in an aggravated inflammatory response and liver damage (37) and eventually led to the death of ducklings. However, the NOD family has long been thought to be involved in antibacterial responses (38–40). Previous studies have shown that only *NOD2* can directly recognize ssRNA viruses (21). More interestingly, there is no *NOD2* gene in poultry (41, 42), which may be related to the complex evolutionary mechanism (43). We speculate that *NOD1* might have replaced the function of *NOD2* and played a role in infection in poultry.

We believe that suppression and overexpression experiments can confirm that the genomic copy number of DHAV-3 is influenced by the *NOD1* expression level. The virus replicates constantly in cells. When the virus was inoculated at an MOI of 1, the virus replication kinetics did not reach a plateau at 30 hpi (44, 45). When the mRNA expression of *NOD1* was suppressed, the DHAV-3 genomic copy number dropped significantly. In contrast, *NOD1* mRNA overexpression resulted in a significant increase in the DHAV-3 genomic copy number. However, due to the lack of a specific and efficient monoclonal antibody against *NOD1* for protein interaction studies, the RNAscope^®^ method was used to observe the localization of DHAV-3 and the RNA levels of *NOD1*-specific markers, confirming that *NOD1* mRNA was widely overexpressed in the DHAV-3-infected cytoplasm. This is not perfect using the same fluorescence to address the colocalization and it is a limit of our study. Further studies are warranted to elucidate the interaction between DHAV-3 and *NOD1* in the future.

The Z8 flock is an artificially selected line with significant resistance against DHAV-3 infection. It cannot be ignored that during the short breeding time of this flock, the selection site could not be accurately located *via* evolutionary analysis. Nevertheless, this information could still be used as an auxiliary means of understanding the resistance of this flock, which is one of the reasons for the formation of traits. Despite that multiple lines of evidence (including from genomic scan and mRNA-seq analyses and DHAV-3 replication and *NOD1* mRNA expression correlation analyses) suggested that *NOD1* was likely involved in the susceptibility of the SZ7 line to DHAV-3 *via* the upregulation of *NOD1* expression that might regulate the expression levels of cytokines (such as *IL8, IFN-β, MAPK1, IFN-γ*, and *IL1-β*) as described previously (Kersse et al., 2011; Moreira et al., 2012; Keestra-Gounder and Tsolis, 2017), however, cytokines are involved in a wide range of physiological activities and *NOD1* doesn't solely and decisively affect their expression, thus, our result is not conclusive, and alternative mechanism(s) might exist, additional investigation is needed to validate our result in greater detail.

## Conclusion

In this study, we showed that *NOD1* was associated with susceptibility to DHAV in ducks. We found that the expression of *NOD1* and its potential downstream genes (including pro-inflammatory factors and interferons) is activated by DHAV-3 infection, which likely results in intensified inflammation and liver damage in ducks. However, further investigation of this mechanism is required. Our results and genomic data provide a target and valuable resource for future investigations of the pathogenic mechanism of DHAV-3 infection and DHAV-3 resistance breeding projects.

## Data Availability Statement

The datasets presented in this study can be found in online repositories. The names of the repository/repositories and accession number(s) can be found in the article/**Supplementary Material**.

## Ethics Statement

The protocols involving animals were approved by the Animal Welfare and Ethics Committee of the Institute of Animal Sciences (IAS), Chinese Academy of Agricultural Sciences (CAAS, Beijing, China) (IAS20160401).

## Author Contributions

SL and SH conceived and designed the experiments. SL performed the experiments. SL, M-SW, and DZ wrote and revised the paper. JT and BZ helped prepare tissue sections. SH, SL, YF, MX, WH, and QZ constructed the Z8 and SZ7 population. All authors read and approved the final manuscript.

## Funding

This work was supported by the National Natural Science Foundation of China (31772592) and the China Agriculture Research System of MOF and MARA (CARS-42).

## Conflict of Interest

The authors declare that the research was conducted in the absence of any commercial or financial relationships that could be construed as a potential conflict of interest.

## Publisher’s Note

All claims expressed in this article are solely those of the authors and do not necessarily represent those of their affiliated organizations, or those of the publisher, the editors and the reviewers. Any product that may be evaluated in this article, or claim that may be made by its manufacturer, is not guaranteed or endorsed by the publisher.
