# Supplementary material for: NOD1 Is Associated With the Susceptibility of Pekin Duck Flock to Duck Hepatitis A Virus Genotype 3
Source: Front Immunol. 2021 Oct 20;12:766740. doi: 10.3389/fimmu.2021.766740 (PMC8563994; doi:10.3389/fimmu.2021.766740)
Supplement: Supplementary file 1 [file DataSheet_1.docx]

**Expression of NOD1 affecting susceptibility of Pekin duck flock to duck hepatitis A virus genotype 3**

Suyun Liang^1,2^, Mingshan Wang^3^, Bo Zhang^1^, Yulong Feng^1^, Jing Tang^1^, Ming Xie^1^, Wei Huang^1^, Qi Zhang^1^, Shuisheng Hou^1*^

1. Key Laboratory of Animal (Poultry) Genetics Breeding and Reproduction, Ministry of Agriculture and Rural Affairs, Institute of Animal Sciences, Chinese Academy of Agricultural Sciences, No. 2 Yuanmingyuan West Road, Haidian, Beijing 100193, China

2. Key Laboratory of Animal Epidemiology of the Ministry of Agriculture, College of Veterinary Medicine, China Agricultural University, Beijing 100193, China

3. Howard Hughes Medical Institute, University of California Santa Cruz, Santa Cruz, CA 95064, USA

*Corresponding author：Shuisheng Hou ([houss@263.net](mailto:houss@263.net))

Mailing address: Institute of Animal Sciences, Chinese Academy of Agricultural Sciences, No. 2 Yuanmingyuan West Road, Haidian district, Beijing 100193, People’s Republic of China.


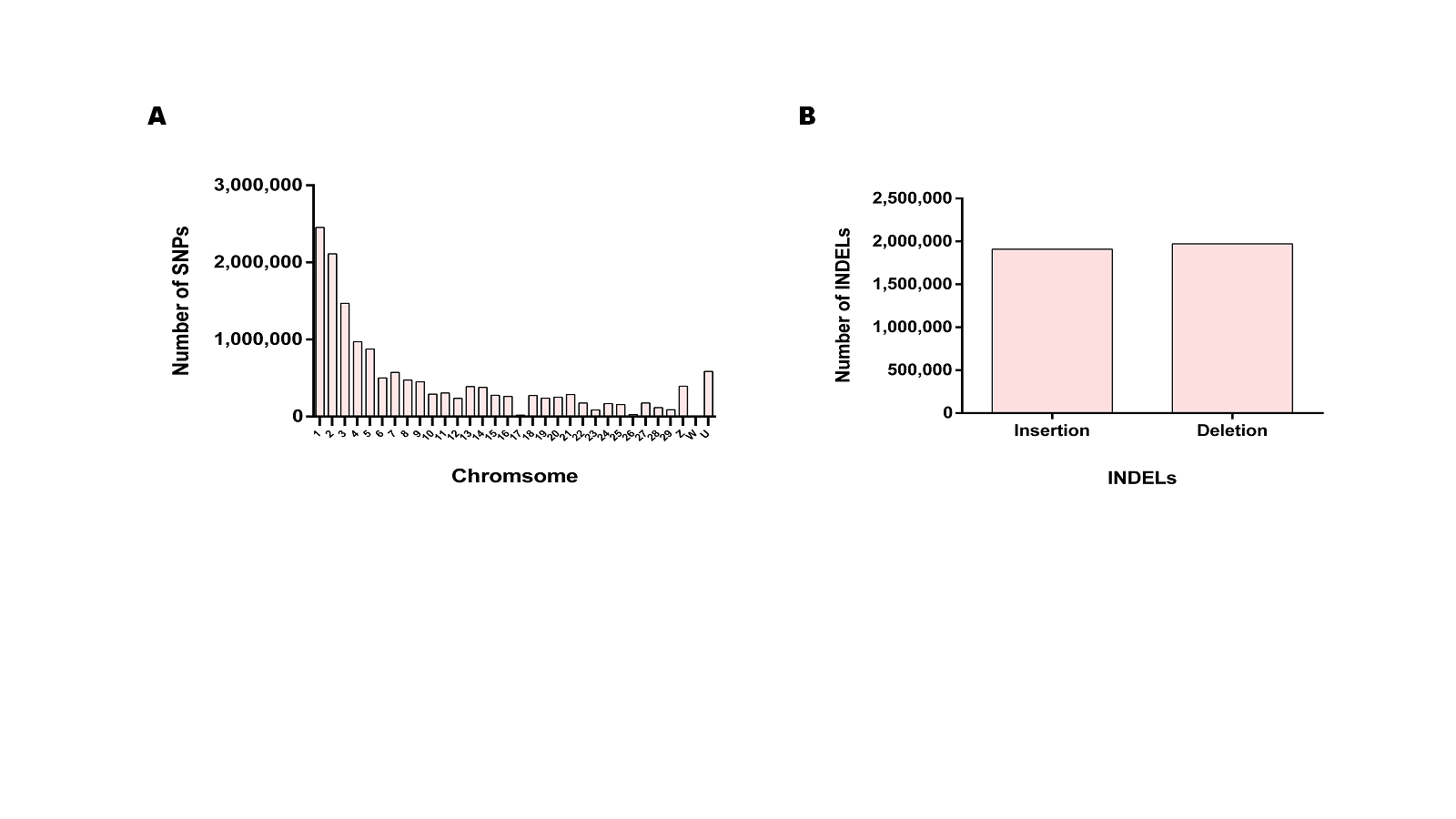


FIG. S1. The variants after aligning to duck reference genome. A. Numbers of SNP. B. Numbers of INDEL.


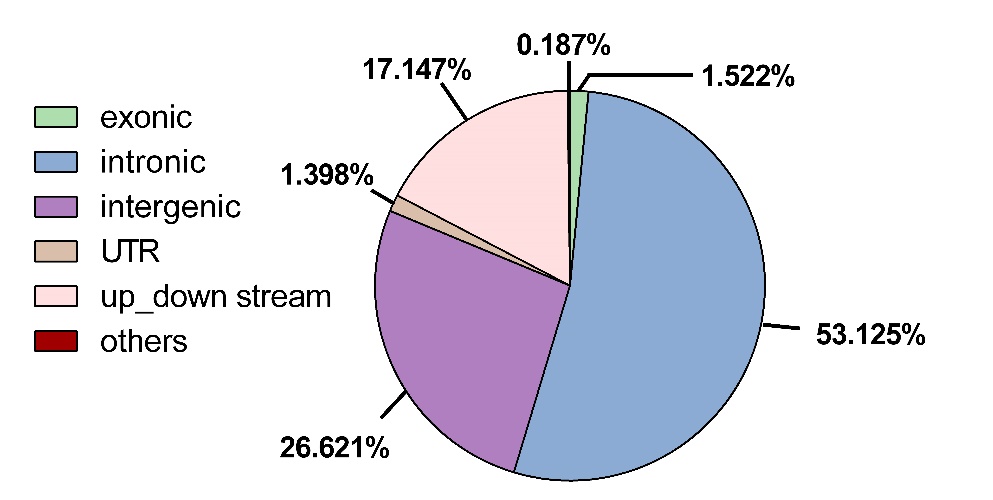


FIG. S2. Overall distribution of variants identified in this study.


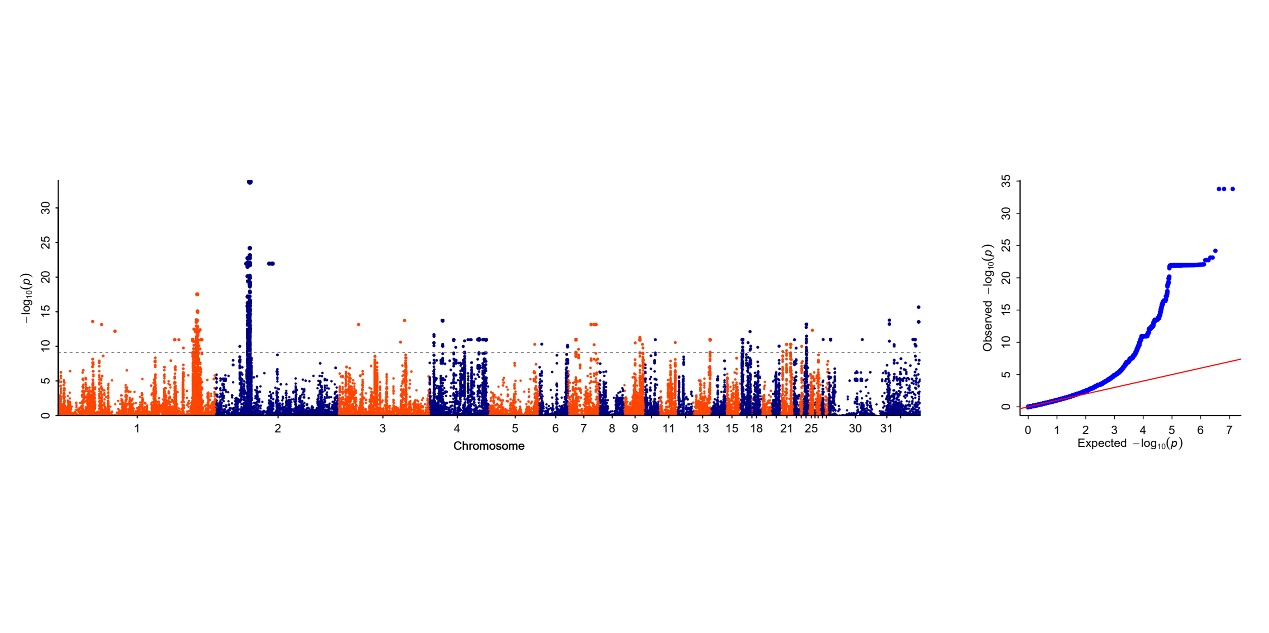


FIG. S3. Manhattan plot and QQ plot of genome-wide association studies used EMMAX. The family number and the first six principal components are fixed effects.


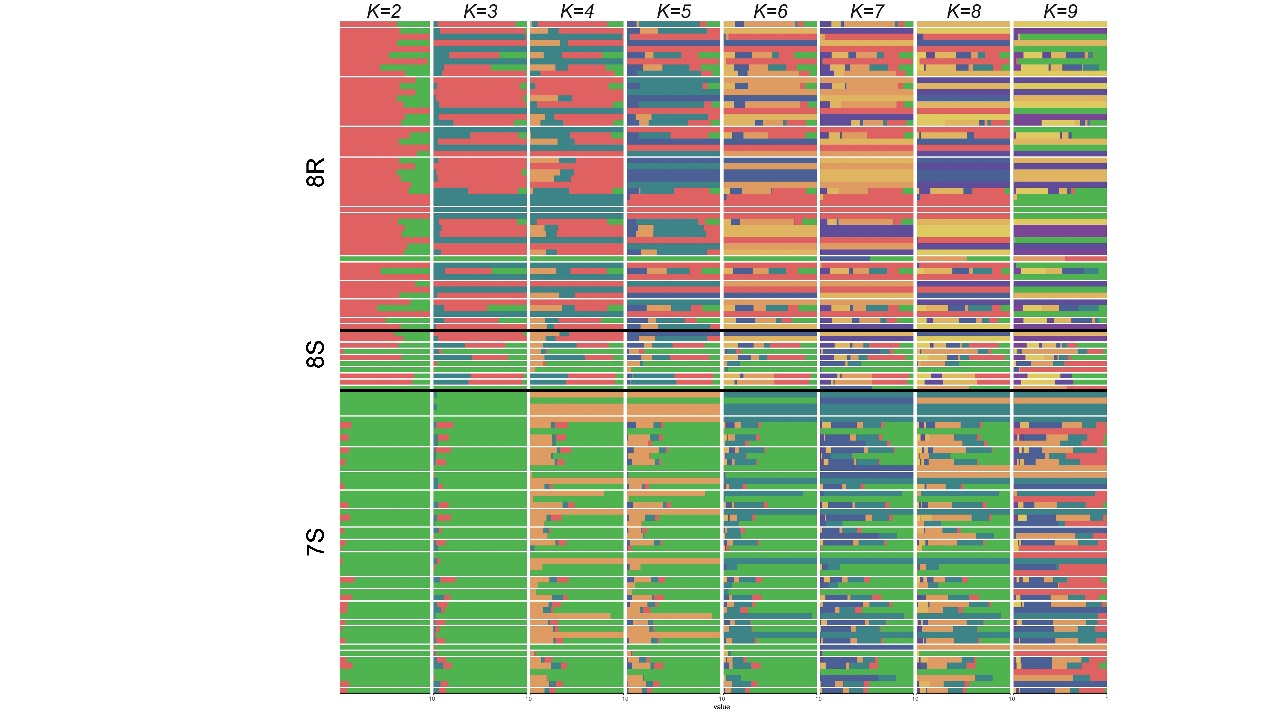


FIG. S4. Admixture analysis for three populations, showing K values from 2 to 9, top to bottom. The analysis is based on autosome chromosomes pruned by PLINK (Chang, et al. 2015).


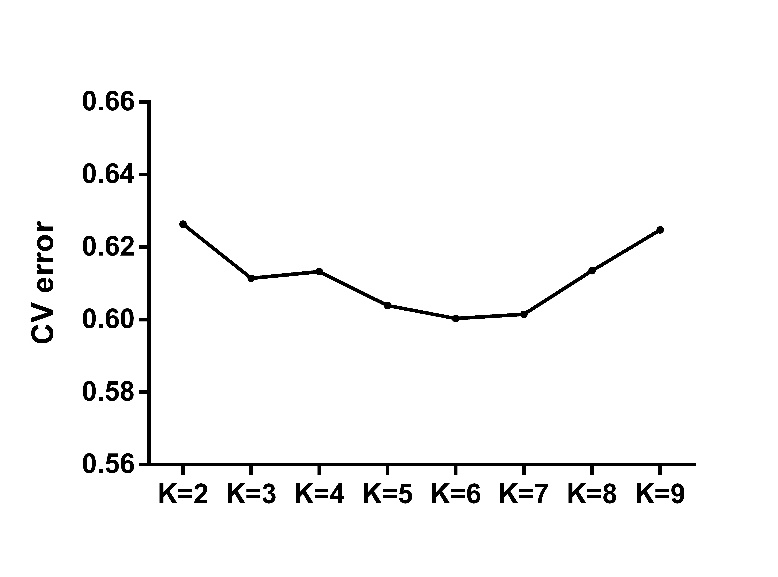


FIG. S5. CV error for the ADMIXTURE analysis at K values from 2 to 9.


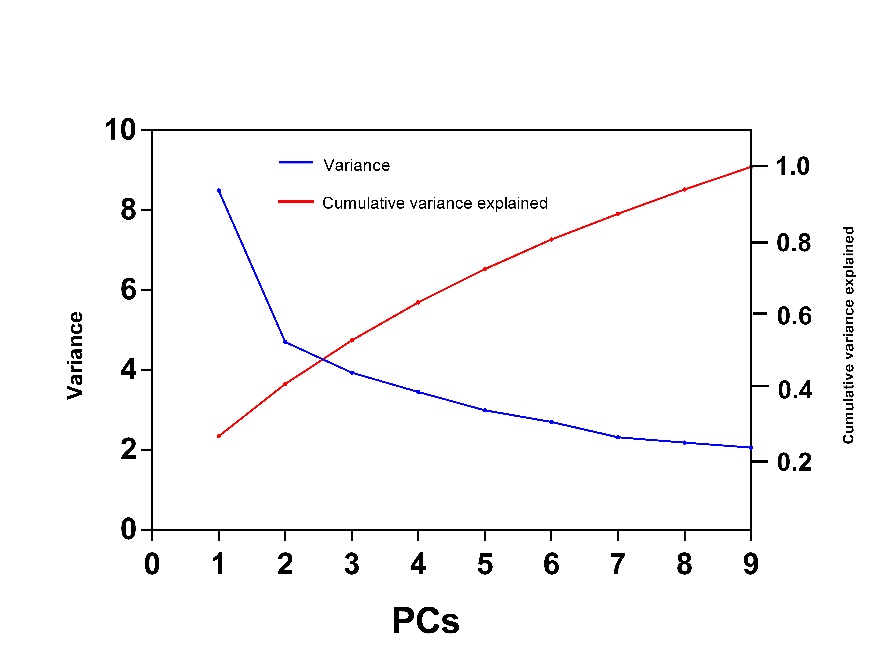


FIG. S6. Principal component analysis for the three populations, generating the genetic relationship matrix from which the first 9 eigenvectors were extracted.


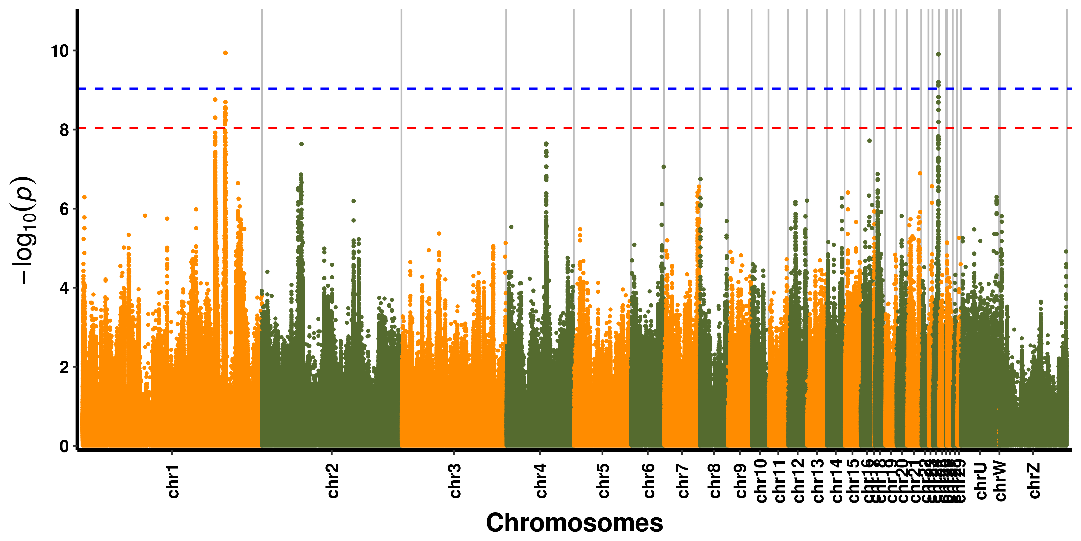


FIG. S7. Manhattan plot of genome-wide association studies used FastLMM.


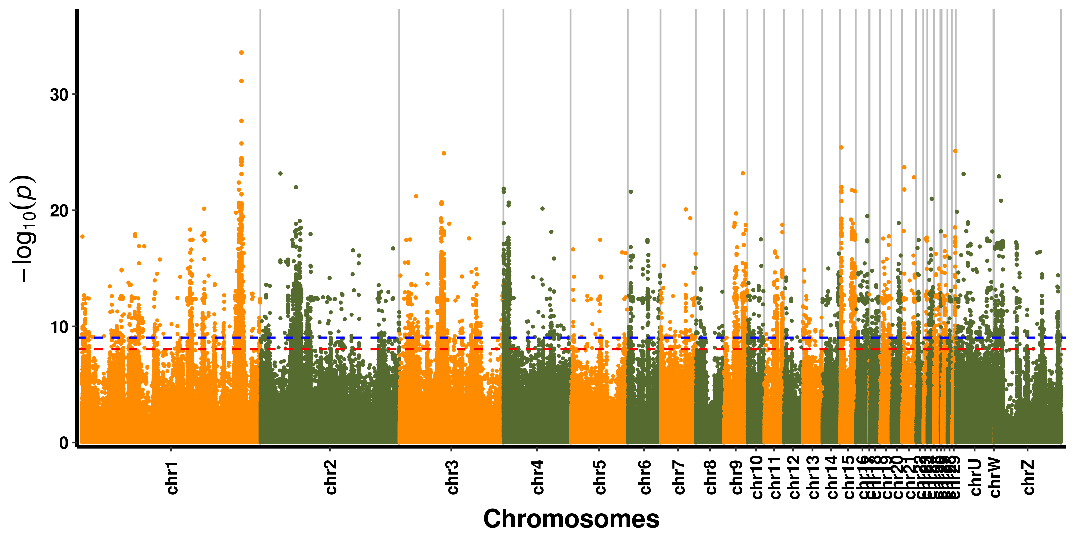


FIG. S8. Manhattan plot of genome-wide association studies used GLM.


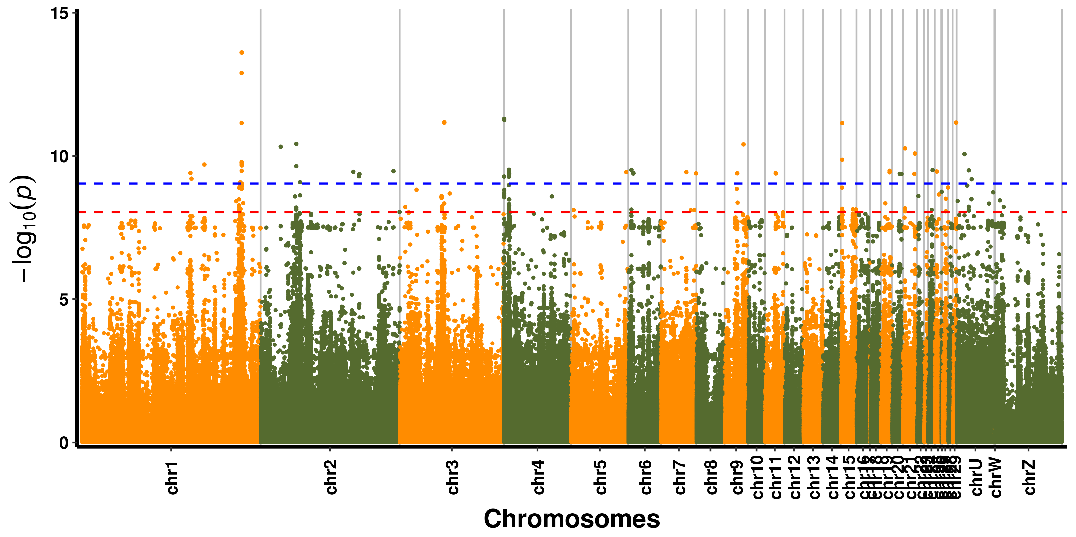


FIG. S9. Manhattan plot of genome-wide association studies used MLM.


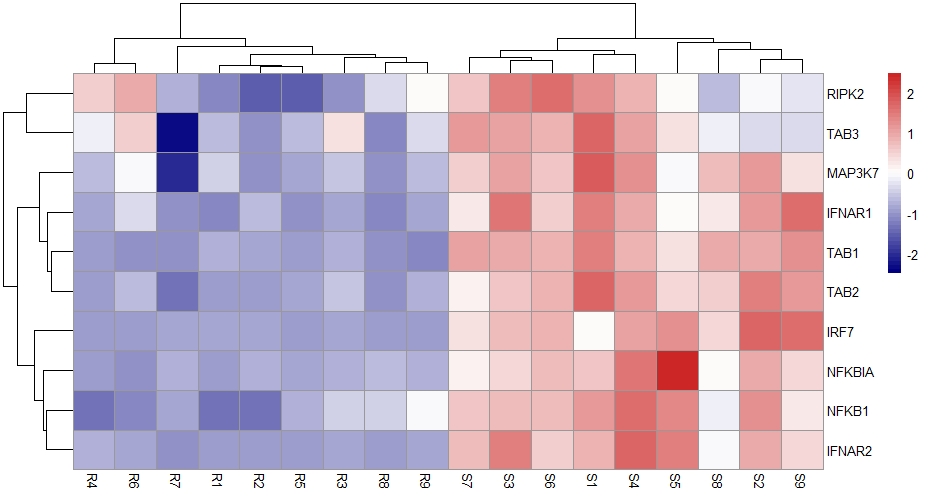


FIG. S10. Gene expression levels of downstream genes.


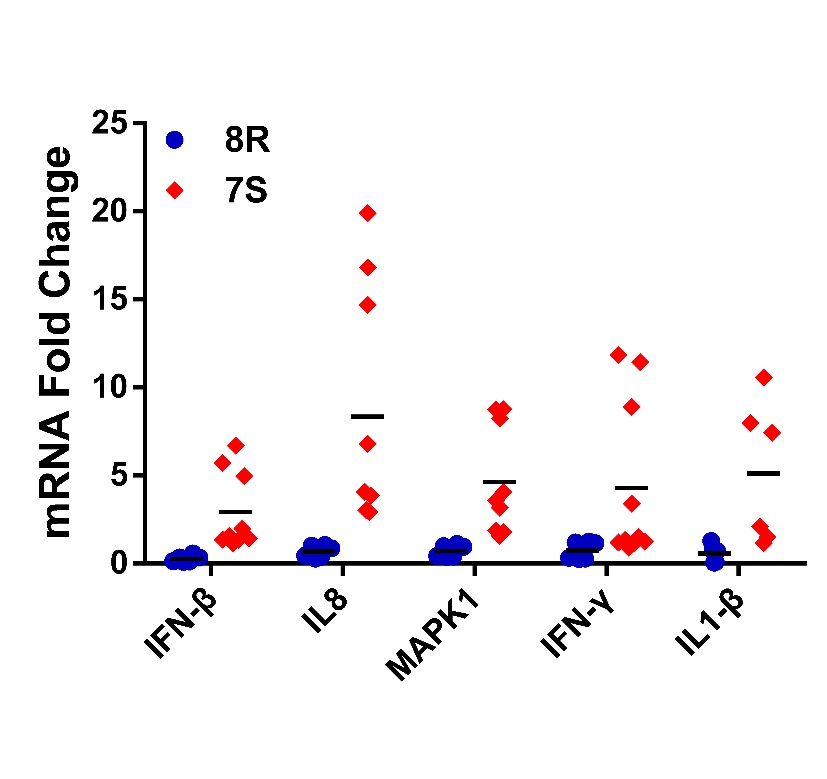


FIG. S11. Results of qPCR for downstream gene expression levels. Each point represents an individual, and each individual has three replicates.


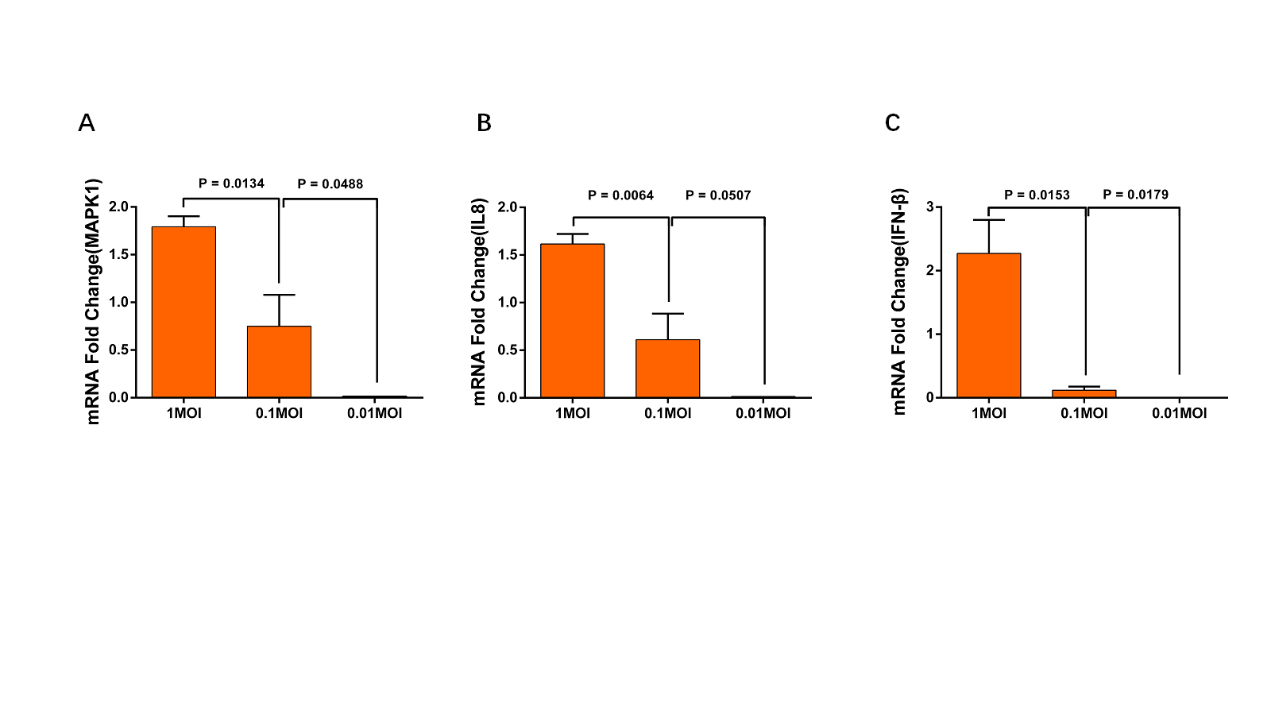


FIG. S12. Results of qPCR for downstream gene expression levels at different MOI. Statistical analysis was performed by Student’s t-test, and error bars indicate standard error of means (SEM).


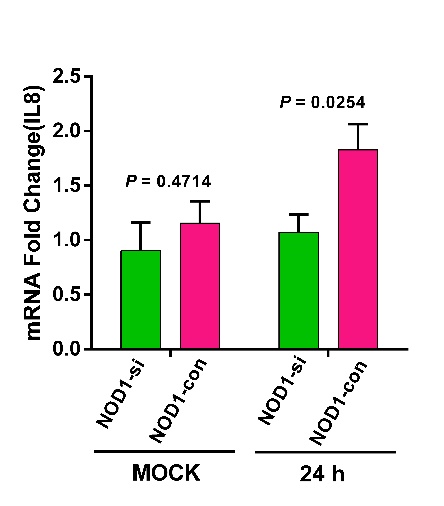

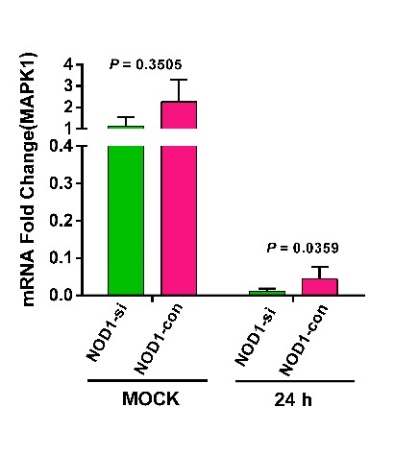


FIG. S13. Results of qPCR for downstream gene expressions after suppression. Results are presented as means ± SD of at least three independent experiments. *, P < 0.05; **, P< 0.01; ***, P < 0.001.


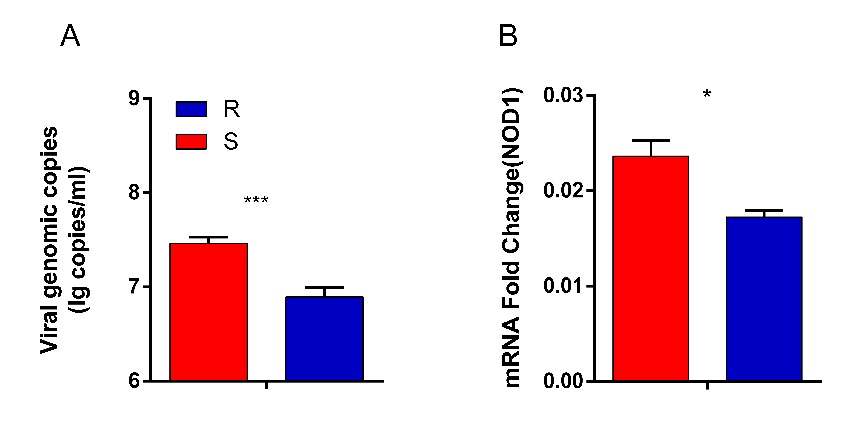


FIG. S14. Results of qPCR for DHAV-3 and NOD1 at 12 hpi. Results are presented as means ± SD of at least three independent experiments. *, P < 0.05; ***, P < 0.001.


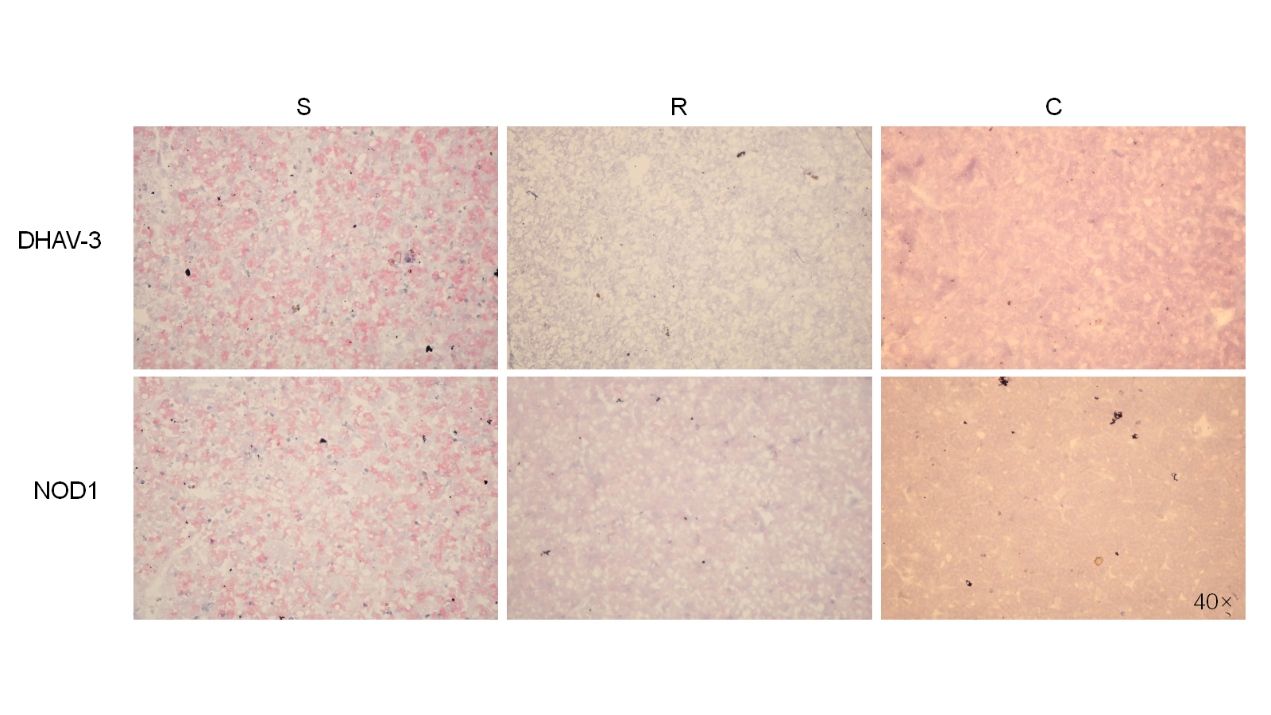


FIG. S15. DHAV-3 and NOD1 were detected by the RNAscope in situ hybridization method. S is the susceptible group, R is the resistant group, and C is the control group. Images were acquired with OLYMPUS microscope. Oil objective: 40×; zoom in 1×.
